# Long-Term Stroke Risk in Patients Undergoing Left Atrial Appendage Ablation With and Without Complete Isolation

**DOI:** 10.3389/fcvm.2021.762839

**Published:** 2021-12-09

**Authors:** Aneesh Dhorepatil, Angela L. Lang, Min Lang, Muhammad Butt, Amit Arbune, David Hoffman, Soufian Almahmeed, Ohad Ziv

**Affiliations:** ^1^Heart and Vascular Institute, Tulane University, New Orleans, LA, United States; ^2^Department of Anesthesia, Critical Care and Pain Medicine, Massachusetts General Hospital, Harvard Medical School, Boston, MA, United States; ^3^Department of Radiology, Massachusetts General Hospital, Harvard Medical School, Boston, MA, United States; ^4^Department of Cardiovascular Diseases, Metro Health Medical Center, Case Western Reserve University, Cleveland, OH, United States; ^5^Division of Cardiology, Gill Heart Institute, University of Kentucky, Lexington, KY, United States; ^6^Heart Associates, Mercy Health St. Elizabeth Youngstown Hospital, Youngstown, OH, United States

**Keywords:** atrial fibrillation, catheter ablation–atrial fibrillation, left atrial appendage, ischemic stroke, left atrial ablation

## Abstract

**Background:** Catheter ablation (CA) for atrial fibrillation (AF), may require ablation beyond the pulmonary veins. Prior data suggest that additional LA ablation, particularly left atrial appendage (LAA) ablation, may alter atrial function leading to increased risk of ischemic stroke or transient ischemic attack (IS/TIA). We sought to study the long-term risk of IS/TIA in patients receiving ablation at the LAA compared to those receiving PVI alone and those receiving PVI with additional non-LAA locations.

**Methods:** 350 patients who underwent CA for AF from 2008 to 2018 were included in the study. Locations of ablation in LA evaluated were the posterior wall, anterior wall, inferior wall, inter-atrial septum, lateral wall and the left atrial appendage (LAA). Patients undergoing LAA ablation were further divided as complete isolation (LAAi) and without complete isolation (LAAa).

**Results:** Mean follow up of 4.8 years. In entire cohort, risk of IS/TIA was 1.62/100 patient-years (pys). The risk was highest in patients with LAAi (3.81/100 pys), followed by ablation LAAa (3.74/100 pys). Amongst all LA locations, only LAAi (HR 3.32, *p* = 0.03) and LAAa (HR 3.18, *p* = 0.02) were statistically significant predictors of IS/TIA after adjusting for OAC (Oral anticoagulant) use and baseline CHA_2_DS_2_VASc score.

**Conclusions:** During long term follow-up, only ablation at the left atrial appendage with and without complete isolation was independently associated with an increased risk of IS/TIA in patients undergoing CA for AF. Potential strategies to reduce stroke risk, such as LAA closure, should be considered in these patients.

## Introduction

Atrial fibrillation (AF) is associated with a 3 to 5-fold increase in the risk of ischemic stroke (IS) (1). The main mechanism associated with the increased thromboembolic risk is thought to be contractile dysfunction and stasis of blood flow (2). Stroke prevention is crucial in AF patients as IS in patients with AF are associated with worse outcomes than IS in patients without AF (3–5). Anticoagulants are effective at reducing thromboembolic risk by about two-thirds, regardless of baseline risks (6–8). For patients with refractory symptomatic AF, catheter ablation (CA) has become an important tool in AF management (9). While pulmonary vein isolation (PVI) remains the mainstay of AF catheter ablation (9), due to disappointing success rates after PVI, various other sites have been targeted for ablation, including the LAA (10). As more of the LA is targeted for ablation, left atrial mechanical dysfunction may develop (11–13) and hypothetically may increase the long-term risk of stroke. These concerns are heightened when dealing with left atrial appendage (LAA) ablation, since the LAA is the source of most AF ablation embolic events in patients with non-valvular heart disease (14–16). Definitive long-term data on the effects of extensive LA ablation on risk of stroke is lacking. In 2016, the BELIEF (the Effect of Empirical Left Atrial Appendage Isolation on Long-term Procedure Outcome in Patients With Persistent or Longstanding Persistent Atrial Fibrillation Undergoing Catheter Ablation) trial found left atrial appendage isolation (LAAi) improved long-term freedom from AF without added complications (17). A subsequent meta-analysis supported the findings, as Romero and colleagues found LAAi was associated with improved recurrence-free survival and was not associated with an increased risk of stroke (18). Two retrospective reports, on the other hand, found a significantly increased risk for ischemic stroke and/or transient ischemic attack (IS/TIA) following LAAi (19, 20). Along the same vein, ablation at the LAA without complete isolation (LAAa), which is at times required during catheter ablation to achieve AF freedom, may also impair LAA contractility and expose patients to similar stroke risk. In the current study, we investigate whether additional ablation beyond the pulmonary veins increases the risk of stroke. We compare long-term stroke risk among patients receiving catheter ablations with ablation at the LAA and those patients receiving catheter ablation without ablation at the LAA.

## Methods

This was a single center retrospective study. IRB approval was obtained prior to initiation of study. Post ablation ischemic Stroke or transient ischemic attack (IS/TIA) was determined by imaging evidence and adjudicated by a neurologist. Paroxysmal AF was defined as a self-limited AF episode lasting < 1 week. Non-paroxysmal AF was defined as a non-self-limiting AF lasting more than 1 week.

Uninterrupted oral anticoagulation (OAC) was continued for 6 months post CA in all patients. After 6 months OAC was continued in patients with CHA_2_DS_2_VASc >2. Anticoagulation discontinuation was per physician's discretion. All Patients with LAAi were recommended lifelong OAC. Patients taking OAC during IS/TIA or at last follow up were described as on OAC and those who were not taking OAC were described as off OAC. Oral anticoagulants used were warfarin, rivaroxaban, apixaban and dabigatran.

A total of 350 consecutive patients undergoing 3D mapping guided radiofrequency CA for AF at a single institution between 2008 and 2018 were included in the study. All procedures were performed with uninterrupted anticoagulation and off antiarrhythmic drugs for a duration equal to 5 half-lives prior to the ablation. Amiodarone was stopped 1 month prior to ablation. An irrigated tip catheter for all procedures with energy delivered from 25 to 40 watts. A 3-D electro anatomical mapping system (Bio sense Webster) was used for guidance during all ablations.

**Figure 1:**
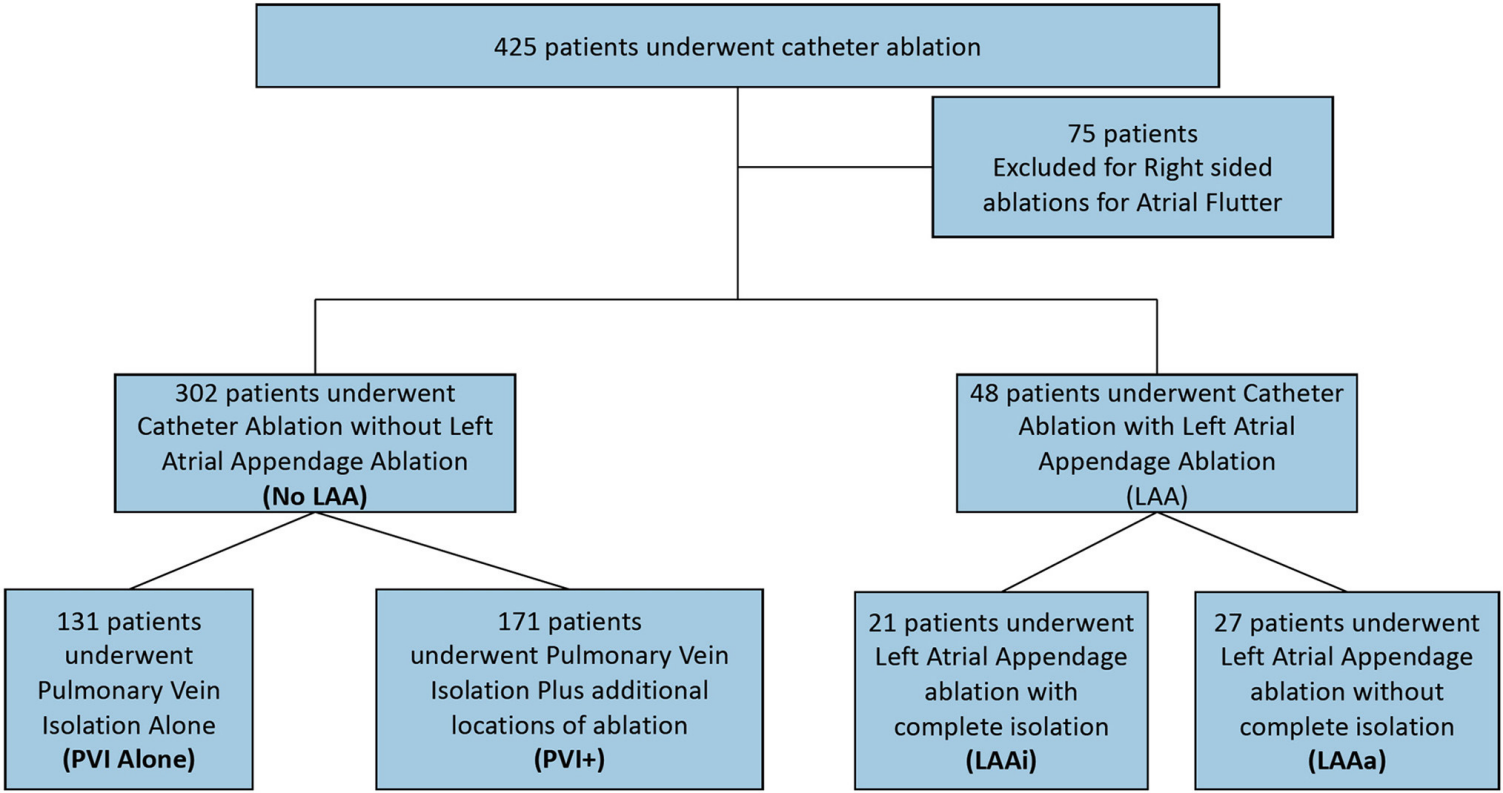
Study design. LAA, Left Atrial Appendage; PVI, Pulmonary Vein Isolation; PVI+, Pulmonary vein isolation with ablation at additional locations except the left atrial appendage; LAAi, Left atrial appendage complete isolation; LAAa, Left Atrial appendage ablation or partial isolation.

Data from patients who had undergone LAA closure or ligation was collected up until the LAA procedure. Seventy-five patients who underwent primarily right sided ablation for atrial flutter were excluded from the study (Figure 1).

Date of follow up was determined as time from last CA until IS/TIA or last follow up. All patients after CA were discharged with an event monitor for the first 3 months set to auto-capture any AF. Thereafter, patients were followed at 3-month intervals for the first year by an electrophysiologist with electrocardiograms at each visit and then once a year. Atrial fibrillation recurrence was defined as presence of AF on electrocardiograms or implantable device reading during IS/TIA or during any office visits 3 months after the last PVI up until the last day of follow up.

**Figure 2:**
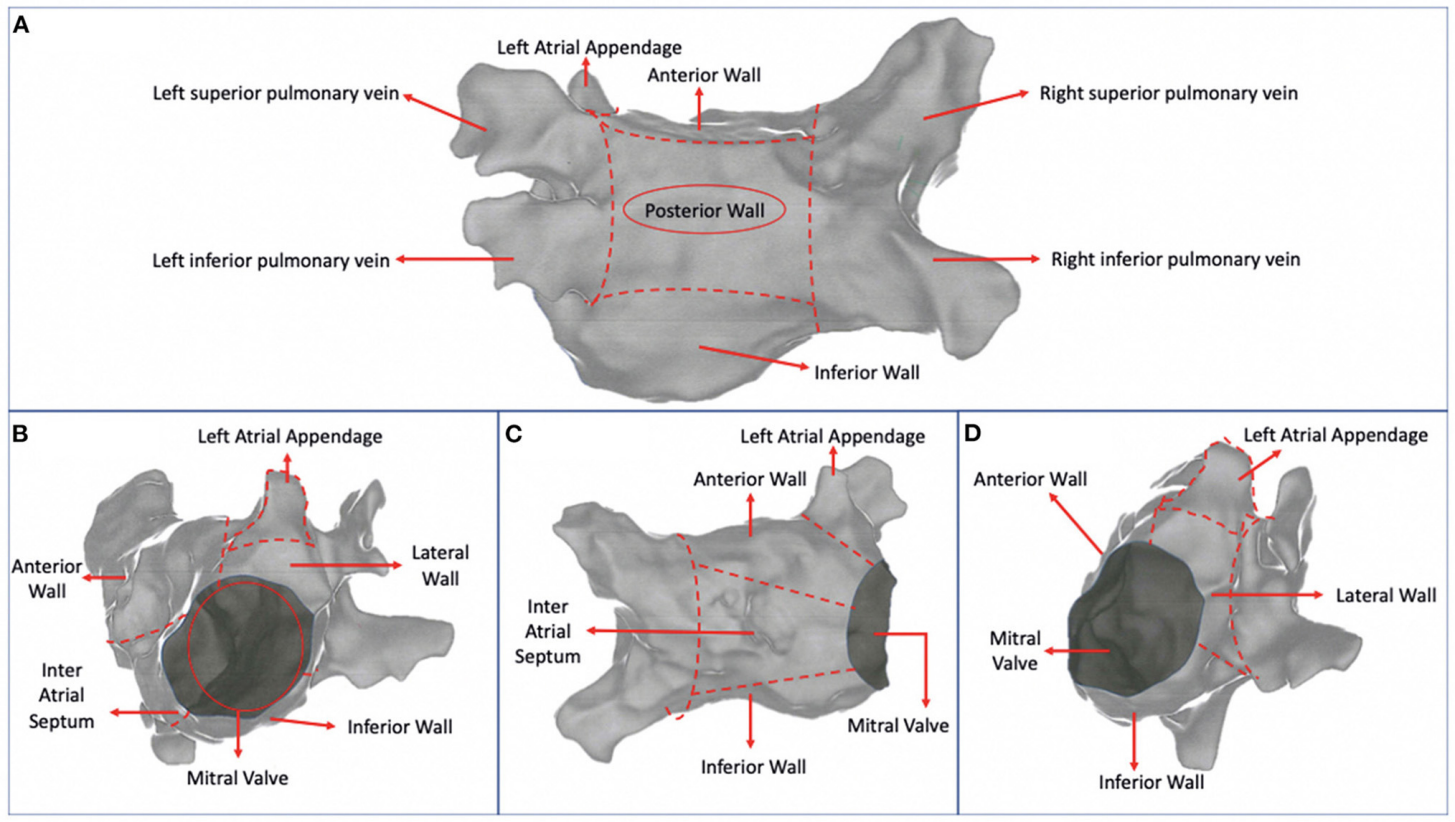
Additional ablation locations.

In patients undergoing CA for AF beyond wide circumferential PVI, multiple locations of ablation in LA were analyzed. These were defined as posterior wall (PW), anterior wall (AW), inferior wall (IW), inter atrial septum (IAS), lateral wall (LW), which included the posterior mitral isthmus and the LAA. The LA was divided into 6 sections using hypothetical lines connecting the contra-lateral pulmonary veins with each other and the superior with the inferior pulmonary veins (Figure 2A). Then each right and left superior and inferior veins were connected with the mitral valve. The PW was defined as the area between the line connecting the superior pulmonary veins and the line connecting the inferior pulmonary veins (Figure 2A). The AW was defined as the area between the line connecting the superior pulmonary veins and mitral valve (Figures 2A–D). The IW was defined as the area between the line connecting the inferior pulmonary veins and the mitral valve (Figures 2A–D). The IAS was the area medial to the line connecting the right pulmonary veins (Figure 2C). The LW included the posterior mitral isthmus and was defined as the area lateral to the line connecting the left pulmonary veins (Figure 2D). The LAA was the area in between the AW and the LW (Figure 2B).

Our primary comparison was between patients undergoing CA excluding the LAA or involving the LAA. Patients undergoing CA excluding the LAA were classified as “No LAA.” To further investigate the role of additional LA ablation in stroke risk, this group was subdivided into patients receiving PVI alone and patients receiving PVI with any additional ablation but excluding the LAA (PVI+). The patients undergoing CA involving the LAA were further divided as either with complete isolation (LAAi) or without complete isolation (LAAa). Isolation of the left atrial appendage was defined either by demonstrating bidirectional block with pacing or with isolated left atrial appendage firing. Patients who underwent LAAa either had either incomplete isolation of the left atrial appendage (failed isolation attempt) or had ablation within the left atrial appendage but without an attempt to completely isolate it. In cases when the left atrial appendage was inadvertently isolated, patients were included in the LAAi group.

### Statistical Analysis

Normally distributed data was presented as mean and standard deviation while non-normal data was presented as median and interquartile (IQ) range. Categorical data was presented as frequencies and percentages. We cross-tabulated categorical variables with chi-square test to determine whether the observed distribution fitted the expected distribution when cell size was sufficient. When the cell size was not enough, Fisher's exact test was used. For continuous covariates, independent sample *t*-test was used to compare the mean.

Non-parametric Kaplan Meier method was used to compare the proportion of patients having IS/TIA in different groups. Survival curves were compared using Log Rank test.

Cox proportional hazards regression analysis was used to estimate the hazard ratio and 95% CI for IS/TIA related to various patient characteristics and types of ablation procedure. Using backward selection and starting with a variable with the largest *P* value, we retained variables that had effect remaining significant in the model at 0.05 in the final model. Statistical analysis was performed with SAS version 9.4 (SAS Inst., Cary, NC). Significance was defined as the 2 tailed value of *P* < 0.05.

## Results

Of the 350 patients, 302 patients underwent CA without LAA ablation of which 131 patients underwent PVI alone and 171 patients underwent PVI+. Forty-eight patients underwent CA involving the LAA of which 21 underwent LAAi and 27 underwent LAAa (Figure 1). On average, 1.47 ablations were required to maintain sinus rhythm. Mean follow up from last CA procedure for all patients was 4.8 ± 2.8 years.

Baseline demographics are listed in Table 1. When comparing patients with “No LAA” to patients “With LAA” all baseline characteristics were similar except the prevalence of non-paroxysmal atrial fibrillation which was more prevalent in the “With LAA” group (70.8 vs. 95.8% *p* 0.0002). Presence of atrial fibrillation at the end of the study was also more prevalent in the ‘With LAA’ group but the difference was not statistically significant (9.9 vs. 18.8% *p* 0.07).

**Table 1:** Baseline demographics^*^.

	**All**	**No**	**With**	***P*** **-value**
	**Patients**	**LAA**	**LAA**	
*N*	350	302	48	-
Age (years)	66.3 ± 10	66.1 ± 10.8	67.9 ± 10.4	0.270
M/F (%)	65/35	66/34	60/40	0.405
Mean follow up (years)	4.8 ± 2.8	4.6 ± 2.7	5 ± 3.1	0.827
HTN (%)	80.1	80.4	79.1	0.833
DM (%)	26.2	25.8	29.1	0.625
CHF (%)	51.1	50.6	54.1	0.651
Prior	10.2	10.5	8.3	0.631
IS/TIA (%)				
Vascular Disease (%)	35.1	35.1	35.4	0.965
Non-Paroxysmal AF (%)	74	70.8	95.8	0.0002
AF at end of study (%)	12.5	9.9	18.8	0.07
CHA_2_DS_2_VASc	2.9 ± 1.7	2.8 ± 1.7	3.1 ± 1.8	0.280
On OAC	67%	65%	78%	0.105

* *HTN, Hypertension; DM, Diabetes Mellitus; CHF, Congestive heart failure; IS/TIA, Ischemic Stroke or Transient ischemic attack; AF, Atrial Fibrillation; OAC, Oral Anticoagulation; LAA, Left Atrial Appendage*.

Of the 302 patients included in the “No LAA” group, 171 patients underwent PVI+. Most common locations ablated were the PW (70%), AW (61%), IW (35%), IAS (31%) and LW (27%). Mean number of additional ablation locations was 1.54.

Forty-eight patients were included in the “With LAA” group. Left atrial appendage ablation with complete isolation (LAAi) was observed in 44% of patients and LAAa was observed in 56% of patients. Most common locations ablated in this group were PW (67%), AW (58%), IW (29%), IAS (33%) and LW (35%). Mean number of additional ablation locations in this group was 1.85.

During the overall 1,665 patient year (pys) follow up, 27 patients (7.7%) had an IS/TIA. The risk of IS/TIA for all patients was 1.62 per 100 pys (Table 2). Of the 27 patients, 13 patients had IS and 14 patients had TIAs. Imaging confirmation was available in 60% (16/27) patients. Sixty percent patients (16/27) were on OAC during the IS/TIA. Prevalence of AF during last follow up was not significantly different in patients with and without IS/TIA (Patients with IS/TIA in AF 25% vs. Patients without IS/TIA in AF 10% *P* = 0.115).

**Table 2:** Ischemic stroke or transient ischemic attack in all patients.

**Type of procedure**	**Risk of IS or TIA**
All patients	1.62/100 pys
No LAA ablation	1.25/100 pys
• PVI alone	1.26/100 pys
• PVI+	1.24/100 pys
LAA ablation	3.77/100 pys
• LAAa	3.74/100 pys
• LAAi	3.81/100 pys

*Risk of IS or TIA per 100 patient years (pys). IS, Ischemic Stroke; TIA, Transient ischemic attack; PVI, Pulmonary Vein Isolation; PVI+, Pulmonary Vein Isolation with additional left atrial ablation; LAA, Left Atrial Appendage; LAAi, Left Atrial Appendage with complete isolation; LAAa, Left Atrial Appendage ablation without complete isolation*.

**Figure 3:**
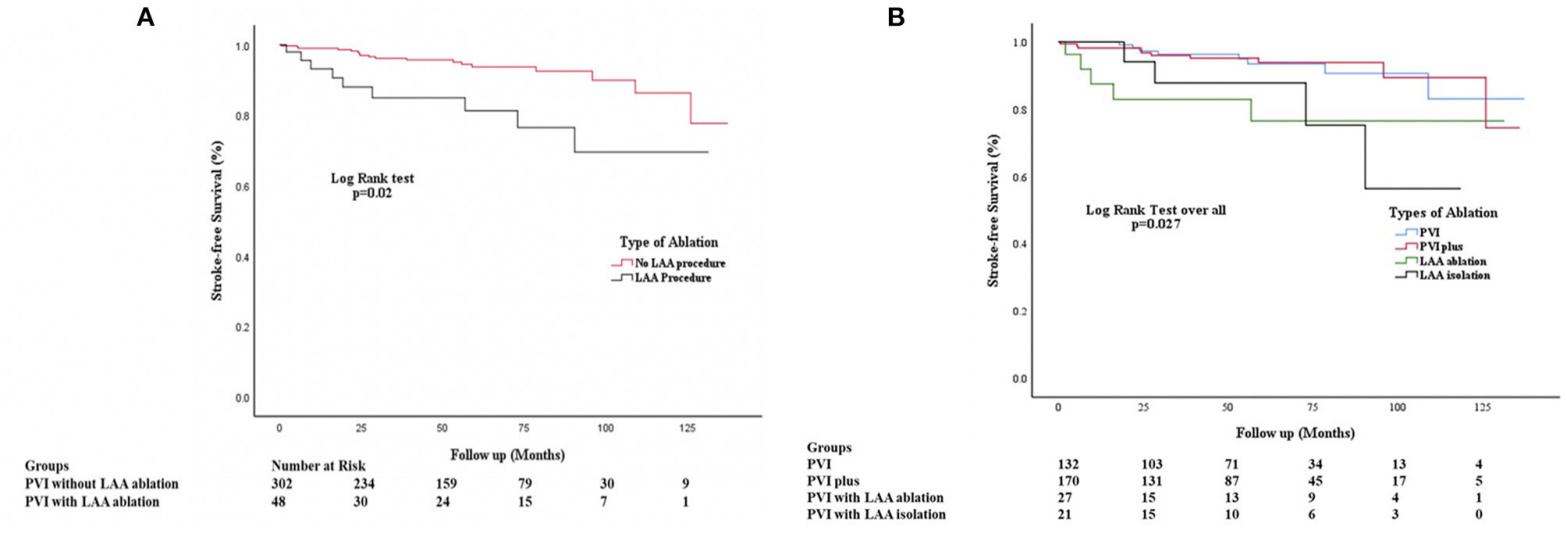
**(A)** Kaplan Meier Stroke-free survival analysis comparing patients undergoing no LAA ablation vs. those who underwent LAA ablation. PVI, Pulmonary vein isolation; PVI+, Pulmonary vein isolation with ablation at additional locations except the left atrial appendage; LAA, Left Atrial Appendage. **(B)** Kaplan Meier Stroke-free survival analysis comparing patients undergoing no LAA ablation vs. those who underwent LAA ablation without complete isolation and with complete isolation. PVI, Pulmonary vein isolation; PVI+, Pulmonary vein isolation with ablation at additional locations except the left atrial appendage; LAA, Left Atrial Appendage; LAAa, Left Atrial appendage ablation or partial isolation; LAAi, Left atrial appendage complete isolation.

The risk of IS/TIA was 3.77 per 100 pys in the patients who underwent LAA ablation and was significantly elevated compared to 1.25 per 100 pys in patients who did not have LAA ablation (HR: 3.14, CI: 1.05–9.4; *P* = 0.003). Kaplan-Meier curves in for IS/TIA free survival are in these are shown in Figure 3A. Kaplan-Meier curves for the subgroups (PVI alone, PVI+, LAAa and LAAi) are shown in Figure 3B. Exploring patients with LAA ablation, 6 patients (67%) were on OAC during the IS/TIA with 3 patients being sub-therapeutic during the IS/TIA (Table 3).

**Table 3:** Ischemic stroke or transient ischemic attack events in patients with Left atrial appendage ablation^*^.

**Sr. No**	**Type of Procedure**	**No. Of Ablations**	**Additional location of Ablation**	**CHA_2_DS_2_VASc** **Prior to event**	**IS or TIA**	**Imaging confirmation**	**OAC during event**	**Reason for OAC** **Discontinuation/interruption**
1.	LAAi	2	None	4	IS	MRI	Off OAC	Bleeding complication with OAC
2.	LAAi	2	None	0	TIA	MRI	Off OAC	Low CHA_2_DS_2_VASc
3.	LAAi	2	IAS	2	TIA	MRI	On OAC	NA
4.	LAAi	2	PW, AW, IW	6	IS	MRI	On OAC	Colonoscopy
5.	LAAa	1	PW, LW	5	TIA	MRI	Off OAC	Bleeding complication with OAC
6.	LAAa	2	PW, AW, IAS	3	IS	MRI	On OAC	Poor compliance
7.	LAAa	2	PW, LW	2	TIA	Neuro	On OAC	Poor compliance
8.	LAAa	1	PW, LW	6	TIA	Neuro	On OAC	None
9.	LAAa	2	PW, AW	6	TIA	Neuro	On OAC	NA

* *IS, Ischemic Stroke; TIA, Transient ischemic attack; PVI, Pulmonary Vein Isolation; PVI+, Pulmonary Vein Isolation with additional left atrial ablation; LAA, Left Atrial Appendage; LAAi, Left Atrial Appendage with complete isolation; LAAa, Left Atrial Appendage ablation without complete isolation; PW, Posterior Wall; AW, Anterior Wall; IW, Inferior Wall; LW, Lateral Wall; IAS, Inter Atrial Septum; Neuro, Neurology confirmed; MRI, MRI Brain confirmation; OAC, Oral Anticoagulation*.

On univariate analysis, ablation at the lateral wall (HR: 1.08 p 0.04) and LAAa (HR: 2.22 *p* 0.04) were statistically significant predictors of an increased risk for IS/TIA (Table 4A). After adjusting for age, gender, CHA_2_DS_2_VASc, OAC status, AF recurrence, other ablation locations in a multivariate analysis using backward selection, both LAAa (HR: 3.18 *p* 0.02) and LAAi (HR: 3.32 *p* 0.03) were statistically significant independent predictors of IS/TIA (Table 4B). No other location of additional ablation in the LA was a significant predictor of IS/TIA. Of note both CHA_2_DS_2_VASc, AF recurrence and OAC status were not significant predictors of IS/TIA in this patient population.

**Table 4:** Univariate and multivariate analysis evaluating risk for ischemic stroke or transient ischemic attack.

(A) Univariate analysis with cox proportional Hazard Ratios evaluating risk of IS/TIA^*^						
**Variable**	**Hazard Ratio**	**Confidence Interval**	***P*** **-value**			
Age	0.98	0.95–1.02	0.302			
Female	1.50	0.70–3.20	0.299			
PW	1.25	0.58–2.69	0.567			
AW	0.91	0.41–2.02	0.810			
IW	0.50	0.15–1.67	0.260			
IAS	0.71	0.24–2.06	0.526			
LW	2.47	1.06–5.78	0.037			
LAAa	2.77	1.04–7.38	0.041			
LAAi	2.81	0.97–8.17	0.057			
AF recurrence	2.19	0.92–5.22	0.077			
AC status	0.74	0.35–1.6	0.450			
CHA_2_DS_2_-VASc	1.08	0.88–1.33	0.450			
Cumulative no. of locations of ablations	1.33	0.93–2.05	0.115			
**(B)** Cox proportional hazards regression model using backward elimination^*^						
**Characteristics**	**Coefficient**	**Standard Error**	**Wald** **χ^2^**	***P*** **-value**	**HR**	**95%** **CI**
LAAa	1.16	0.51	5.17	0.02	3.18	1.17 to 8.64
LAAi	1.20	0.56	4.67	0.03	3.32	1.10 to 9.84
(C) Effect of each additional LA ablation location in patients without LAA ablation using univariate cox proportional hazards regression Model^*^						
**Characteristics**	**Coefficient**	**Standard Error**	**Wald** **χ^2^**	***P*** **-value**	**Hazard Ratio**	**95%** **CI**
Number of LA locations ablated	−0.087	0.18	0.22	0.64	0.92	0.64 to 1.32

* *LAAa, Left Atrial Appendage Ablation without complete isolation; LAAi, Left Atrial appendage ablation with complete Isolation; PW, Posterior Wall; AW, Anterior Wall; IW, Inferior Wall; LW, Lateral Wall (including Posterior Mitral Isthmus); IAS, Inter Atrial Septum.*

We further explored whether ablation at multiple locations was associated with risk of IS/TIA. Patient who underwent LAA ablation were excluded in this analysis as LAA ablation was already shown to be associated with stroke risk. Cox proportional hazards regression analysis showed that there was no statistically significant increase in the risk of IS/TIA in patients undergoing ablations at multiple sites (HR: 0.92, CI: 0.64–1.32, *p* 0.64) (Table 4C).

## Discussion

Ablation at the LAA for AF has been associated with improved AF free survival but may come at the cost of increased risk of stroke (19, 20). The peculiar anatomy and physiology of the LAA has been hypothesized to lead to increased risk of IS/TIA through multiple studies (2, 21–23). Even in the absence of ablation or isolation, atrial myopathy may lead to LAA dysfunction, which increases the thrombogenicity of the LAA (24). The current study demonstrates two important findings. First, ablation at the left atrial appendage regardless of whether complete isolation was achieved was associated with an increased risk of stroke in long term follow-up. Interestingly this increase in risk was independent of CHA_2_DS_2_VASc or OAC status. Second, in patients who received extensive additional ablation that did not include the left atrial appendage, long term risk of stroke was not elevated compared to patients undergoing PVI alone.

Ablation of non-pulmonary vein sites for AF is frequently used to try and improve AF free survival (10). However, the long-term risk of IS/TIA in these patients has so far not been thoroughly investigated. In the current study, the risk of IS/TIA in patients undergoing PVI+ was not significantly elevated when compared to PVI alone (1.24/100 pys compared to 1.26/100 pys, respectively). Furthermore, other than the LAA, no other location of ablation was an independent predictor of future stroke. Finally, cumulative ablations beyond the pulmonary veins that did not include the LAA did not increase the long-term risk of stroke. Further studies in the form of prospective studies will be needed to assess the risk of stroke in these patients.

The thrombogenicity of ablation at the left atrial appendage remains controversial. In the BELIEF trial (17) authors found that LAAi was an effective procedure to reduce recurrence rates of AF at 24 months. Interestingly, no patients in the LAAi group had an IS/TIA but 4.5% of patients in the standard ablation group had an IS during the 24 months follow up. Since its publication, there has been increasing concern regarding the risk of stroke post procedure. Rillig et al. (19) conducted retrospective study with 50 patients undergoing LAAi which showed a 6% risk of IS/TIA and a 21% incidence of LAA thrombus over a 12 month follow up. The largest long-term outcome study was by Kim et al. (20) which showed an increased risk of IS/TIA in patients undergoing LAAi when compared to PVI. Furthermore, they showed a 23% risk of IS/TIA in patients undergoing LAAi over 5 years of follow up. This current study similarly demonstrated that LAAi and LAAa were independently associated with an increased risk of IS/TIA with a rate of 3.8/100 pys and 3.7/100 pys, respectively.

The increased stroke risk post-LAAi and LAAa may be related to the method of LAAi and post ablation residual LAA function. Recently, Di Biase et al. (25) showed that patients with LAAi with impaired LA function and suboptimal OAC were at an increased risk of IS/TIA. Interestingly, they were also able to show that in patients with preserved LAA function after LAAi, there was no increased risk of stroke following the procedure. The risk of IS/TIA in the current study was higher than those reported by Di Biase and colleagues (1.7/100 pys compared to 3.8/100 pys, and 3.7/100 pys). This perhaps could be secondary to the increased anticoagulation rates in their study. However, it is important to note that in patients who had LAA ablation but no isolation, lifelong anticoagulation is not currently standard of care. Our study suggests that these patients may need to be treated in the same manner as LAAi patients. Furthermore, although our patients with LAAi were recommended lifelong anticoagulation, our long-term real-world follow-up shows that interruption in anticoagulation due to non-cardiac procedures or patient non-compliance is not uncommon. Therefore, we raise concern about long term sustainability of uninterrupted oral anticoagulation in all patients. Our study suggests that prior to performing LAAi or LAAa, a physician must carefully counsel the patient on the need of uninterrupted anticoagulation lifelong. Finally, our data suggests that in some patients IS/TIA risk may be elevated despite uninterrupted OAC, since 67% of the patients post LAA ablation had an event on OAC and only half of these patients had suboptimal OAC. Other groups have found a similar concerning pattern. Riling et al. and Kim et al. reported that up to 50% of patients (post LAAi) with events were on OAC (19, 20). This increased risk despite OAC may be due to persistent mechanical dysfunction of the LAA and increased thrombogenicity within the LAA.

This increased risk despite OAC may be secondary to persistent mechanical dysfunction of the LAA or an increased thrombogenicity within the LAA. Increased levels of plasma Von-Willebrand factor (vWF) have been associated with an increased risk of stroke (26, 27). Patients with non-valvular AF have increased levels of immunoreactive vWF within the LAA which could lead to increased thrombogenicity (28). Recently this increase in vWF was also demonstrated in patients undergoing minimally invasive surgery for atrial fibrillation (29). Left atrial appendage isolation or ablation leads to endocardial damage at the left atrial appendage, which could lead to increased vWF, and consequently increasing the *in-situ* risk of LAA thrombi formation. Further research in this area will be needed.

The risk of stroke in patients undergoing ablation at the LAA, raise the question of whether LAA closure has a role in preventing stroke in these patients. Di Biase et al. (25), reported that in patients with impaired LAA function after LAA isolation LAA closure or ligation was performed with good clinical outcomes. Conversely, in this cohort, patients with preserved LAA function were able to stop OAC without increased stroke risk during the 2 year follow up. In our patient population, strokes occurred in our post LAA ablation patients in patients with and without uninterrupted anticoagulation. Our study raises the question of whether prophylactic closure (30, 31) of the LAA after isolation or ablation is reasonable since both lead to an increase risk of stroke in long term follow-up. This can be achieved percutaneously via an endocardial approach using closure devices or via an epicardial approach to excise the LAA.

Currently there are multiple percutaneous closure devices which utilize either a nitinol-based cage (Watchman^TM^) or self-expandable plugs (Amulet^TM^) (32). These approaches have large datasets supporting their use for stroke prevention in the AF population, but less data is available on patients with LAA isolation or ablation. However, in smaller studies have demonstrated that LAAO in patients with LAAi is a safe (33) and effective procedure for preventing IS/TIA (25). A hybrid (endo-epi) approach using the LARIAT system achieves electrical isolation and LAA ligation utilizing an endo-epicardial suture delivery system (34). This novel technique has a high success rate of LAA isolation (35), but large long term stroke prevention data has not been published (36, 37). Early concerns about procedural complications, predominantly related to bleeding (36, 38) have been reduced with further refinement of the technique (37, 39). The ongoing aMAZE trial (40) (percutaneous alternative to the Maze procedure for the treatment of persistent or long-standing persistent atrial fibrillation)which is randomizing patients with long standing persistent AF to PVI with LAA ligation (using LARIAT) vs. PVI alone, will shed further light on the safety and efficacy of this approach.

Surgical approaches to LAA exclusion involve either LAA ligation or LAA exclusion using devices (41, 42). Usually these are performed concomitantly with cardiac surgery. Complete surgical excision has been demonstrated to be more effective in long term closure but requires an open chest procedure (43). Use of exclusion devices allow for minimally invasive approaches that could be good alternatives to percutaneous LAA exclusion. A thoracoscopic approach using the AtriClip device has been shown to achieve LAAi and LAA exclusion simultaneously with minimal complications (44). While it has been shown to be an effective strategy for LAA exclusion, large long-term data regarding IS/TIA prevention and comparison with established LAA occlusion strategies is lacking (35). Randomized studies will be needed to explore the efficacy, safety, and feasibility of such a strategy.

### Limitations

The current study has several limitations. This study was retrospective in nature. Although we attempted to account for multiple confounders, there may be underlying differences in our groups that accounted for the differences in stroke risk that we measured. While our follow-up duration is long, nearing 5 years, our cohort size is relatively small compared to some prior studies (17, 19, 25). Despite the small size of the cohort, the robust follow-up in all subgroups allowed us to demonstrate significant increase in stroke risk in patients with LAA ablation. We believe that in patients with LAA ablation with and without isolation, LAA mechanical dysfunction is the cause of increased stroke risk. However, we did not investigate LAA function in our patients and therefore do not have a mechanistic explanation for our observations. In our cohort 39/48 (82%) patients undergoing LAAa or LAAi also had ablation at the other locations in the LA. While only LAA ablation was an independent predictor of stroke, it is possible that additional ablation locations when added to LAA ablation increase stroke risk. This possible interaction may need further investigation. Finally, we did not differentiate between modes of LAA ablation. It is possible that some more circumferential LAA ablation is less likely to raise the long-term stroke risk by preserving LAA function. It is important to note that LAA residual function post isolation would be dependent on automaticity in the LAA. The reliability of such automaticity is unknown and may require more long-term investigation to fully understand whether residual LAA function can fully mitigate stroke risk.

## Conclusion

Catheter ablation of LA sites beyond the pulmonary veins can be useful in AF free survival. The current study suggests that additional ablation that excludes the LAA, is not associated with an increased risk of stroke when compared to PVI alone. LAA ablation is an emerging field, and more data is required to identify optimal management and improve safety. Our study also suggests that patients with ablation in the left atrial appendage, regardless of whether complete isolation was achieved or not, have an elevated long-term risk of stroke. For, these patients uninterrupted OAC, therefore, should be offered regardless of the CHA_2_DS_2_VASc score. It is also important to recognize that despite continuation of OAC, these patients may have elevated risk of stroke. The use of prophylactic LAA closure in these patients should be explored.

## Data Availability Statement

The raw data supporting the conclusions of this article will be made available by the authors, without undue reservation.

## Ethics Statement

The studies involving human participants were reviewed and approved by MetroHealth IRB. Written informed consent for participation was not required for this study in accordance with the national legislation and the institutional requirements.

## Author Contributions

AD: conceptualization, methodology, project administration, data curation, writing-original draft preparation, writing-review and editing, and supervision. AL and ML: writing-original draft preparation, formal analysis, and verification. MB: writing-review and editing, formal analysis, and software. AA and DH: conceptualization. SA: investigation, resources, and writing-review and editing. OZ: conceptualization, methodology, project administration, writing-original draft preparation, writing-review and editing, supervision, investigation, and resources. All authors contributed to the article and approved the submitted version.

## Conflict of Interest

The authors declare that the research was conducted in the absence of any commercial or financial relationships that could be construed as a potential conflict of interest.

## Publisher's Note

All claims expressed in this article are solely those of the authors and do not necessarily represent those of their affiliated organizations, or those of the publisher, the editors and the reviewers. Any product that may be evaluated in this article, or claim that may be made by its manufacturer, is not guaranteed or endorsed by the publisher.
